# Puppyhood diet as a factor in the development of owner‐reported allergy/atopy skin signs in adult dogs in Finland

**DOI:** 10.1111/jvim.16211

**Published:** 2021-07-14

**Authors:** Manal B. M. Hemida, Siru Salin, Kristiina A. Vuori, Robin Moore, Johanna Anturaniemi, Sarah Rosendahl, Stella Maria Barrouin‐Melo, Anna Hielm‐Björkman

**Affiliations:** ^1^ Department of Equine and Small Animal Medicine Faculty of Veterinary Medicine, University of Helsinki Helsinki Finland; ^2^ Department of Nutrition and Clinical Nutrition Faculty of Veterinary Medicine, Beni‐Suef University Beni‐Suef Egypt; ^3^ Department of Agricultural Sciences Faculty of Agriculture Forestry, University of Helsinki Helsinki Finland; ^4^ Department of Veterinary Anatomy Pathology and Clinics, School of Veterinary Medicine and Zootechny, Federal University of Bahia Salvador Brazil

**Keywords:** DogRisk, early, fish, food, hygiene, immune, life, meat, microbiota, organs, questionnaire, raw, tripe, unprocessed

## Abstract

**Background:**

The increased prevalence of atopic dermatitis (AD) in dogs necessitates research in its disease etiology.

**Objectives:**

To explore the association between puppyhood dietary exposures and prevalence of owner‐reported allergy/atopy skin signs (AASS) after the age of 1 year.

**Animals:**

Four thousand and twenty‐two dogs were eligible, 1158 cases, and 2864 controls.

**Methods:**

This cross‐sectional hypothesis‐driven observational study was extracted from the DogRisk food frequency questionnaire. Forty‐six food items and the ratio of 4 major diet types were tested for their association with AASS incidence later in life. Potential puppyhood dietary risk factors for AASS incidence were specified using binary multivariable logistic regression. The model was adjusted for age and sex.

**Results:**

Eating raw tripe (odds ratio, 95% confidence intervals OR, 95% CI = 0.36, 0.16‐0.79; *P* = .01), raw organ meats (OR, 95% CI = 0.23, 0.08‐0.67; *P* = .007), human meal leftovers, and fish oil supplements as well as eating more that 20% of the diet as raw and/or <80% of the diet as dry, in general, were associated with significantly lower AASS incidence in adulthood. In contrast, dogs fed fruits (OR, 95% CI = 2.01, 1.31‐3.07; *P* = .001), mixed‐oil supplements, dried animal parts, and dogs that drank from puddles showed significantly higher AASS incidence in adulthood.

**Conclusions and Clinical Importance:**

Puppyhood exposure to raw animal‐based foods might have a protective influence on AASS incidence in adulthood, while puppyhood exposure to mixed oils, heat processed foods and sugary fruits might be a potential risk factor of AASS incidence later. The study suggests a causal relationship but does not prove it.

**Abbreviations**
AASS: allergy/atopy skin signs
AD: atopic dermatitis
BPA: bisphenol A
DHA: docosahexaenoic acid
*E. coli*
: *Escherichia coli*
EPA: eicosapentaenoic acid
FFQ: food frequency questionnaire
Ig: immunoglobulin
IL: interleukins
LA: linoleic acid
MR: Maillard reaction
*n*‐3 PUFA: long‐chain omega‐3 polyunsaturated fatty acids
*n*‐6 PUFA: omega‐6 polyunsaturated fatty acids
Th: T helper (cells)

## 1 INTRODUCTION

Atopic dermatitis (AD) in dogs is usually defined as an inflammatory allergic skin disease that begins during the first 3 years of life.^1^, ^2^ The worldwide AD prevalence in dogs was estimated to be approximately 10%‐15% in 2001.^3^ In 2017, an owner‐reported AD prevalence of 18.3% was reported in an earlier version of our own questionnaire data.^4^ This high prevalence makes exploring the disease etiology and possible preventive measures important, as although AD affects dogs with genetic predispositions for the disease, genetics alone cannot justify the increased prevalence over the last few years.^2^, ^3^, ^4^

Early life environmental exposures, such as diet have been found to impact the development of the immune system and subsequently disease susceptibility later in life in both animals and humans alike.^5^, ^6^ This was first proposed by Strachan^7^ who suggested that a lack of microbial exposure during childhood explains the increased prevalence of allergic diseases. Such exposures are supposed to be essential for programming the immune system and modifying its future inflammatory responses.^8^ In light of the hygiene hypothesis, a reformulated hypothesis, known as the “microflora hypothesis,” has been proposed. It argues that early life exposures to beneficial nonpathogenic microbes can alter the individual's microbiome development, influence the innate and adaptive immune system, and cause permanent consequences for the individual's health.^9^, ^10^

Deworming the dams during pregnancy, sunlight exposure, normal body condition score, born in the same family where the dog lives, and exposure to rural environment have all been found to decrease owner‐reported AD prevalence.^11^ Additionally, other genetic and background factors such as the maternal history of owner‐reported AD, age, sex, breed, and color have previously been studied.^11^ In the study on the modifiable early risk factors for AD development in dogs, we reported that maternal and puppyhood diets based on nonprocessed meat were significantly associated with a decreased prevalence of owner‐reported AD, while a diet based on ultraprocessed carbohydrate‐rich foods were significantly associated with an increased risk for owner‐reported AD.^11^ The nonprocessed meat/ingredients at early life may lead to microbial exposure that enhances the immune system's maturation which thus may help mitigate allergies.^11^ A study on humans reported that the consumption of unpasteurized milk during childhood was associated with a lower prevalence of AD when compared with the consumption of pasteurized milk.^12^ Research that show the similarities between canine and human AD, including the increased prevalence, clinical manifestations, diagnostic standards,^5^ and host‐microbiome interaction,^6^ highlight that the dog is an ideal model for studying, for example, AD pathogenesis.^6^

The aim of the current study was to investigate the association of puppyhood dietary exposures with later owner‐reported allergy/atopy skin signs (AASS) incidence using a broad range of food items reported by the owners in a food frequency questionnaire.

## 2 MATERIALS AND METHODS

### 2.1 Study design

The DogRisk food frequency questionnaire (FFQ; http://www.ruokintakysely.fi/) is an epidemiological, owner‐reported cross‐sectional questionnaire established in 2009 at the University of Helsinki to inspect the incidence of the noncommunicable diseases in dogs in relation to different dietary and nondietary variables. Details of the FFQ and its validation have been previously published.^4^, ^11^, ^13^ The questionnaire was ethically approved (29.4.2016) by the Viikki campus ethical board, University of Helsinki.

### 2.2 Data curation and dogs

**FIGURE 1:**
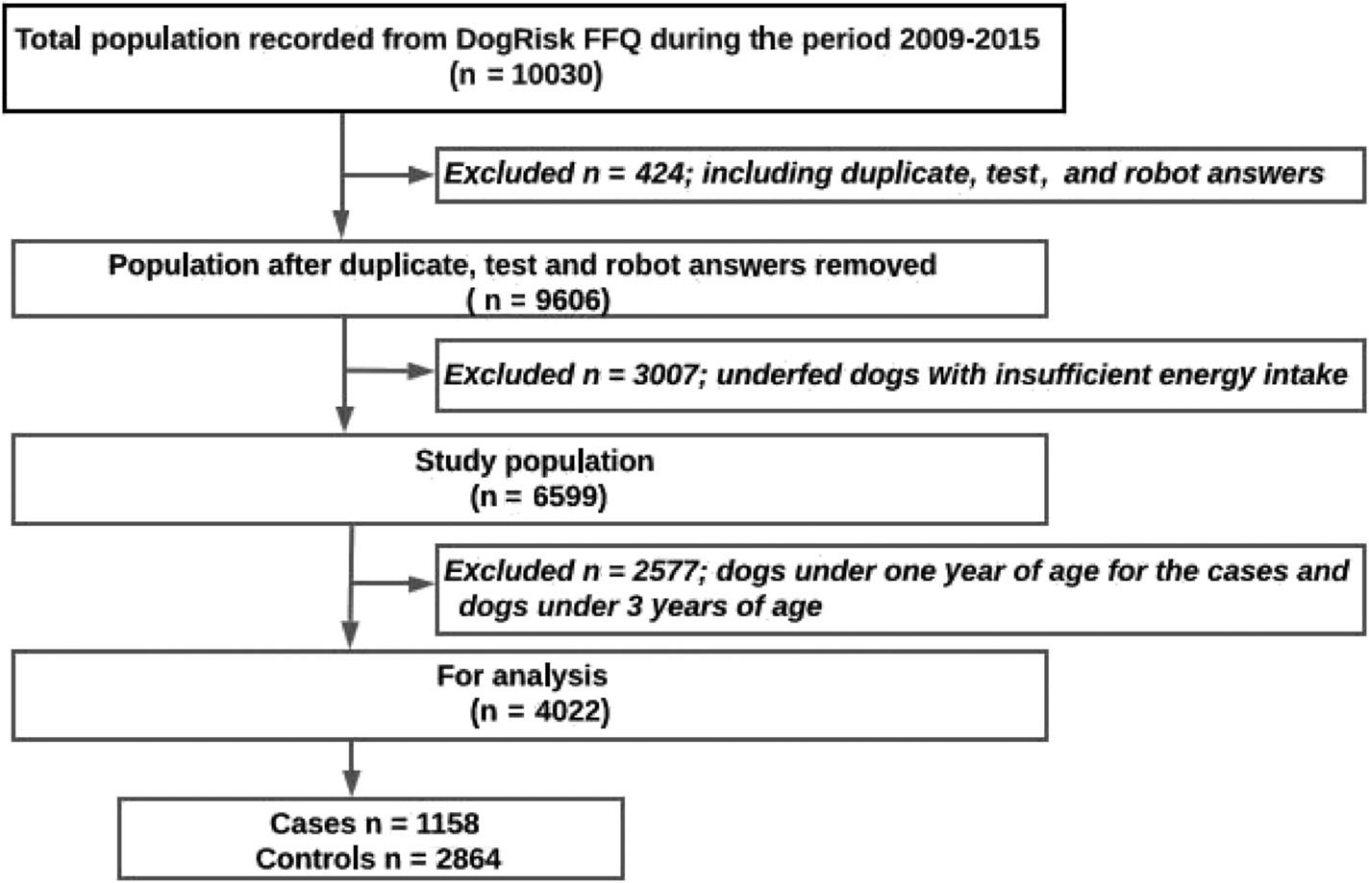
Flowchart of the study sample. FFQ, food frequency questionnaire

The data were cleaned by removing duplicates, test, and robot answers (Figure 1). A sum variable on energy‐containing feeds (sources of protein, fat, and carbohydrates) was calculated for each dog at consecutive age periods and this estimate was used as an eligibility criterion. The nonquantitative FFQ included both open‐ended and drop‐down menu questions. Hence, the frequency of food items consumption could be indicated by the owner in different sections. To avoid overestimation of intake frequencies of the food items, the original data were reduced by clustering the identical food items with similar nutrient profiles and processing methods into a total of 53 categories. There were also quantitative data on the percentage of the dogs' diet which determined their categorization to 4 different feeding patterns. If the total diet percentage (miscalculated sums) exceeded or was below 100%, the numbers were handled as follows: the extreme values of the unadjusted totals (<90 and >110) were excluded. The unadjusted totals (100 ± 10) were adjusted to 100%.

As shown, 10 030 dog owners' responses were screened for the study's eligibility criteria (Figure 1). Dogs that had enough energy dense food at least 5 days per week and that had an answer to the AD related question (Table S2, Supporting Information) were eligible. In order to prevent reverse causality, dogs under the age of 1 year were excluded from the case group.^11^, ^14^ As AD in dogs usually occurs during the first 3 years of life, dogs under 3 years of age were excluded from the control group. The remaining 4022 dogs were included for statistical analysis (Figure 1).

### 2.3 Study variables

The dependent variable (binary outcome) was the answer to a question on owner‐reported AD status (Table S2, Supporting Information). The true binary question reflecting AD in the questionnaire was “Allergy/atopy producing skin signs” (AASS) (no/yes) in their dogs. The “No” answers were categorized as controls, while “Yes” were counted as cases of AASS. We referred to the disease as AASS in the current study. There were 6 other dermatology/pruritus related diagnoses before and after the AASS option that the owner could choose from: “Other skin infection, eg, hot‐spot,” “seborrhea,” “demodicosis,” “furunculosis,” “acanthosis nigricans,” and “otitis.” The study variables are reported in detail in Supporting Information Table S1 and the binary AD question as well as the other dermatological diagnoses the owner could choose from, are shown in Supporting Information Table S2.

In the present study the owners were asked to answer the following question regarding the consumed ratio of different feeding patterns by providing answers in percentages that added up to 100%. The question was: Could you please estimate how many % of your 2‐8 months old dog's food was: (A) raw food, (B) dry food, (C) other commercial dog foods, and (D) home‐cooked food? A total of 5617 responders were selected after these adjustments were made.

Additionally, the study screened 46 food items, indoor/outdoor edibles, that were ingested by dogs during the age of 2 to 6 months for the association with the future development of AASS. The independent food variables/predictors were analyzed as categorical ordinal variables, each containing 5 categories of frequency of ingestion: 0 = “never,” 1= “a couple of times per year,” 2= “a couple of times per month,” 3= “a couple of times per week,” and 4= “daily or almost daily.”

### 2.4 Statistical analysis

The data were analyzed using SPSS (SPSS statistics for Windows, version 25.0, IBM Corp, Armonk, NY). Odds ratios were visualized using the “forest plot” v. 1.10^15^ package for R (Package “forestplot,” version 1.10, in R software, version 4.0.1).^16^ The prevalence of AASS within adult dogs in relation to the different consumed ratios in different feeding patterns was calculated by crosstabulation. Associations between predictors and outcome variables were determined by chi‐square tests (likelihood ratio) with Bonferroni correction to adjust *P*‐values for multiple testing and to reduce type I errors. Liberally associated variables (*P* < .20) were accepted into multivariable modeling (Supporting Information Table S1). Multivariable logistic regression was run to calculate the odds ratios (OR), 95% confidence intervals (CI), of the association between the independent variables screened previously (with *P*‐values <.20) with the binary dependent variable. Backward stepwise regression including 36 variables with entry probability ≤.05 and removal probability ≥.1 was used for modeling. The model was adjusted for age and sex and the missing values were not imputed. The variables with *P* < .05 were considered statistically significant. The final model fit quality was determined by the Omnibus test (*P*‐value <.05), Hosmer and Lemeshow test (*P*‐value >.05) and Nagelkerke's *R* value, which should be as large as possible.^17^, ^18^

## 3 RESULTS

### 3.1 Study sample characteristics

The study dogs characteristics are presented in Table 1. The dog breeds were categorized into allergy prone breeds and nonallergy prone breeds based on data from a previously published study.^11^ The prevalence of owner‐reported adult AASS in the study dogs was 17.6%.

**TABLE 1:** Characteristics of the studied dogs divided into allergy/atopy in dogs cases and controls

Items	Cases (n = 1158; 28.8%)	Controls (n = 2864; 71.2%)	Total study dogs (n = 4022; 100%)
*Age/years, median (min., max.)*	4 (1; 17)	5 (3; 17)	5 (1; 17)
Missing	0; 0	0; 0	0; 0
*Sex*			
Males	544; 47	1276; 44.6	1820; 45.3
Females	610; 52.7	1581; 55.2	2191; 54.5
Missing	4; 0.3	7; 0.2	11; 0.3
*Breed*			
Nonallergy prone breeds	541; 46.7	1763; 61.7	2304; 57.4
Allergy prone breeds	617; 53.3	1096; 38.3	1713; 42.6
Missing	0; 0	5; 0.1	5; 0.1
*Color*			
Less white	567; 49	1498; 52.3	2065; 51.3
≥50% white	288; 24.9	742; 25.9	1030; 25.6
≥90% white	255; 22	461; 16.1	716; 17.8
Missing	48; 4.1	163; 5.7	211; 5.2

*Notes*: Values are presented in numbers and ratios unless otherwise mentioned.

### 3.2 Impact of the consumption of different feeding patterns during puppy age on the prevalence of AASS in adult age

**FIGURE 2:**
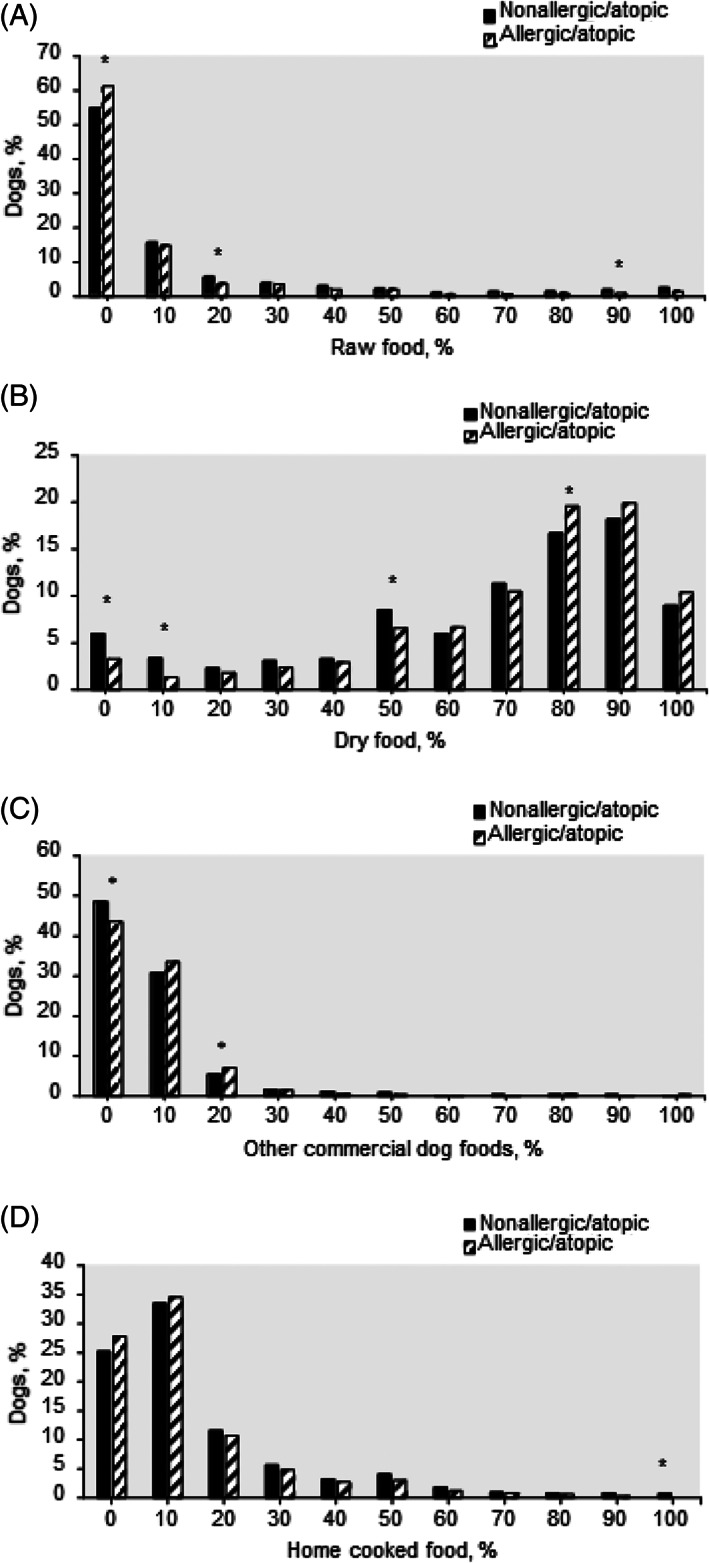
Proportions of nonallergic/atopic and allergic/atopic dogs within puppies (from 2 to 6 months of age, n = 5617) consumed different feeding patterns at different ratios. A, raw food, n = 4962; B, dry food, n = 5544; C, other commercial dog foods, n = 5125; D, home‐cooked food, n = 5242. *, the difference between nonallergic/atopic and allergic/atopic dogs' percentage is significant at *P* < .05

The prevalence of adult AASS within the group of dogs for which we had information of their puppy diet at 2 to 6 months of age differed in the consumed ratio of raw food, dry food, other commercial dog food, and home cooked food (Figure 2). Most of the dogs consumed a mixture of these 4 diet groups. Consumption of at least 20% of the diet as raw food (Figure 2A), or below 80% of the diet as dry food (Figure 2B) were significantly associated with a decreased prevalence of AASS in dogs, while no consumption (zero %) of raw food or 80% or more of dry food significantly associated with an increased prevalence of AASS in dogs. Similarly to dry food, also other processed commercial dog food (Figure 2C) significantly associated with a decreased incidence of AASS when not fed at all (zero %), while they associated with an increased prevalence of AASS in dogs when consumed at 20%. The significance in the latter, as well as in the home‐cooked food group (Figure 2D) is debatable due to a very low number of dogs in the significant groups.

### 3.3 Association of puppyhood food variables with adult AASS incidence in the study sample

From the contingency table (Table S1) showing the frequencies of the covariates within the study cases and controls, we found that there were 27 out of 46 puppyhood food variables, that were significantly associated with the incidence of AASS at adult age.

**FIGURE 3:**
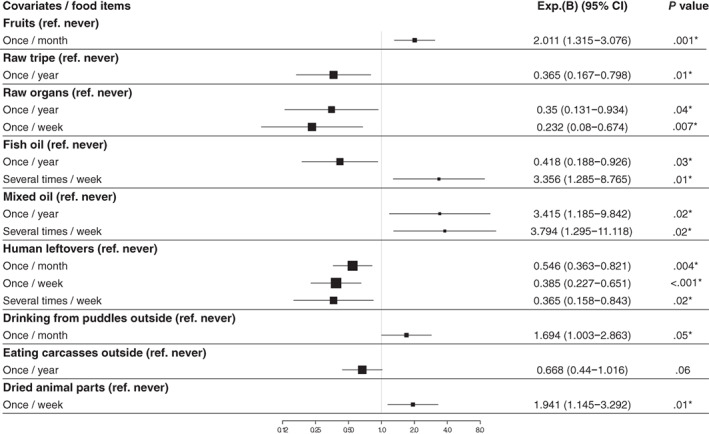
Odds ratios of the association between the puppyhood diets and the incidence of allergy/atopy skin signs in adult dogs based on multivariate logistic regression analysis (n = 4022). The model was adjusted for age and sex. The number of the puppies included in the multivariate logistic regression model were 957 dogs. Exp.(B), the odds ratio (OR) based on the multivariate logistic regression analysis using backward stepwise deletion method; CI, confidence interval; *, the significant association with *P* ≤ .05; ref, reference

The results from the multivariable logistic regression model are shown in Figure 3. Eight variables were significantly associated with the incidence of AASS later in life: Eating raw tripe (odds ratio, 95% confidence intervals [OR, 95% CI] = 0.36, 0.16‐0.79; *P* = .01), raw organ meats (OR, 95% CI = 0.23, 0.08‐0.67; *P* = .007), human meal leftovers, and fish oil supplement during puppyhood was associated with a significantly lower AASS incidence in adulthood. In contrast, dogs fed fruits (OR, 95% CI = 2.01, 1.31‐3.07; *P* = .001), mixed oil supplement, dried animal parts (OR, 95% CI = 1.94, 1.14‐3.29; *P* = .01), and dogs that drank from outside puddles during puppyhood showed a significantly higher AASS incidence in adulthood. The results are presented in Figure 3.

## 4 DISCUSSION

The key finding from the present dog sample‐based study was the significant association between the puppyhood diet (from 2 to 6 months of age) and the tendency to develop AASS in adulthood. Studies on dogs have shown that AD results from interactions between the individual's genetics, epigenetics, immune system response and environmental allergens, especially diet.^2^, ^11^ It has been widely assumed that early life dietary exposures might be a major contributor to the development of AD in dogs later in life.^11^ Furthermore, it has been reported that gene expression may constantly be epigenetically regulated by the early diet,^19^, ^20^, ^21^ via promoting gut microbiota diversity.^22^ Recent studies have demonstrated that the composition of the gut environment, including both its diversity in microbes and chemical compounds, differs between healthy and AD patients.^23^, ^24^, ^25^

Therefore, recognizing the possibly protective and risky foods for AASS introduced during the puppyhood period would be useful for designing early preventive measures to lessen AASS incidence among dogs, which we discuss next in light of our results.

### 4.1 Puppyhood dietary exposures that might lower AASS incidence in adulthood

Our results showed that introducing raw meat‐based diets (here raw tripe and raw organs meats) to puppies at 2 to 6 months with different frequencies (Figure 3) associated negatively and significantly with AASS incidence in adulthood. These findings support our previous observation that the consumption of a nonprocessed meat‐based diet during puppyhood may protect against AD in dogs later in life.^11^ Interestingly, in an epidemiological study on 106 dogs, Sallander et al^26^ found that feeding puppies noncommercial homemade diets that included minced beef from 2 to 6 months of age was significantly associated with a higher risk of CAD incidence, but this was true only if the dam was not fed homemade diets including meat, egg and milk products during lactation. When the dam was fed what the authors called noncommercial animal products during lactation, there was no difference in AD risk for the offspring, regardless if puppies were fed meat or not. ^26^ The previous study highlights the importance of the diet of the dam, especially among high‐risk breeds for CAD.

To the best of our knowledge there has been no scientific research conducted on raw tripe as a dog food previously. The potential protective effect of raw tripe on AASS incidence is credited to its abundance in the living beneficial bacteria such as *Lactobacillus acidophilus*, making raw tripe a “functional food” and a good source of natural probiotics for dogs.^27^, ^28^ Probiotic bacteria have a profound effect on establishing the basis for intestinal flora development and modulation in the early ages^29^ and their functions provides protection against AD via multiple biological pathways.^24^, ^30^ It is known that AD is associated with an imbalanced ratio of T helper cells (Th1/Th2) with a predominance of proinflammatory interleukins IL‐4, IL‐5, and IL‐13.^31^ Exposure to probiotics at an early age provides beneficial long‐term clinical and immunomodulation effects in humans^32^, ^33^, ^34^, ^35^ and in an AD canine model^30^ by decreasing the allergic and inflammatory factors via modulating the gut‐skin axis,^32^ and modulating the stress response from the brain through the gut‐brain‐skin axis.^36^ Probiotics can also boost mucosal homeostasis by displacing gut pathogens by *Lactobacillus* species^37^ and initiating mucin production.^38^

Our study also showed that eating raw organ meats during puppyhood was a significant and possibly protective factor against AASS incidence in adulthood. Raw organ meats refer to raw carcass offal such as liver, heart, kidneys, lungs, tongue, and so forth. Raw organ meats are dense in vitamins, minerals and trace‐minerals and are also a good source of high‐quality protein and fat (USDA food composition database).^39^ Since some of these nutrients are heat‐sensitive, especially the vitamins,^40^ offering organs raw to puppies allows them to get the maximum nutritional value. Moreover, raw organ meats as a raw meat‐based foods are a good source of beneficial bacteria which may help puppies develop a healthy gut microbiota.^41^, ^42^

Human meal leftovers offered to the puppies was found to be significantly associated with less AASS later in life. The possibly protective effect increased with its frequency, hence the more exposure puppies had to human meal leftovers, the more protection against AASS development there was. The literature on the benefits or risks of feeding pet dogs leftovers from human meals, is poor. Finnish cuisine and feeding habits over the past years have transitioned toward a higher consumption of healthy fresh foods, while the consumption of sweets, sugar and soft drinks has decreased.^43^, ^44^ Traditionally popular Finnish dishes are composed of fish and meats, vegetables and roots, mushrooms, buttermilk and other fermented milk products, berries, and the whole grain products, for example, black rye bread and oatmeal.^45^ The raw meats scraps (pork, beef, organs, fish, and chicken) and the trimmings of the extra fatty parts covering the meat, might modulate and favor a more diverse gut microbiome.^41^, ^42^ Moreover, roots, vegetables and whole grain products are rich in various indigestible and soluble fibers, which act as dietary prebiotics.^46^, ^47^, ^48^, ^49^, ^50^ Prebiotics are fermented in the colon by enteric bacteria to produce short chain fatty acids that alter the gut microbiota. This may offer advantages for the enteric epithelium and host's general health.^51^, ^52^, ^53^ Feeding leftover sour and fermented dairy products may help maintain a balanced inflammatory response through the presence of living microbes which may help improve the intestinal microbial balance.^54^, ^55^, ^56^ Moreover, healthy humans might share various mouth resident microbial species with their dogs,^57^ that are transferred to the puppies by the mutual contact with table utensils and hands. These oral microflorae, like *Streptococcus salivarius*, can inhibit cytokine expression in epithelial cells.^58^ Furthermore, offering table scraps to dogs as a kind of petting is a strong indicative of a more intense human‐canine bond, which may decrease the stress a puppy experiences and affects the immune system positively in both dogs^59^, ^60^, ^61^ and their owners.^62^

Feeding puppies, a fish oil supplement was significantly associated with lower AASS incidence when “given a couple of times per year,” while giving it “always or almost always” became a potential risk factor for development of AASS later in life. Fish oil is rich in the long‐chain omega‐3 polyunsaturated fatty acids (*n*‐3 PUFA), eicosapentaenoic acid (EPA) and docosahexaenoic acid (DHA), which are known for their beneficial effects toward the management of multiple inflammatory diseases including AD in dogs.^63^ Moreover, the *n*‐3 PUFA has been found to be positively correlated with anti‐inflammatory activity.^64^ Evidence from human epidemiological and intervention studies concluded that there is a protective association between fish oil supplementation during infancy and atopic outcome.^65^ However, from the current study findings, it was seen that the overconsumption of fish oil supplement when given “always or almost always” increased the risk of AASS. Similarly, studies have demonstrated that the effects of EPA and DHA on the innate immunity are dose‐dependent in canines^66^ and humans.^67^ Furthermore, some authors have argued that unbeneficial effects on immune function are more likely possible when EPA and DHA are given in large amounts.^68^, ^69^, ^70^ Clinical studies are required to further elucidate the correct prophylactic dosage of oils in dogs, given as potential protection against AASS.

### 4.2 Puppyhood dietary exposures that might increase AASS incidence in adulthood

Eating fruits during puppyhood was significantly and positively associated with AASS incidence in later life. Surprisingly, many owners reported that they often offer their dogs canned fruits, which usually contains added sugar. The sugariness of newly bred fruit's cultivars has also continued to increase.^71^ High sugar intake early in life induces metabolic dysregulation and gut inflammation that results in negative alterations in gut microbial communities.^72^, ^73^ Fructose, that is, the fruits' predominant sugar, has been linked with several detrimental health issues^74^, ^75^ including low‐grade inflammation which plays a key role in chronic diseases pathogenesis.^76^ Stimulating the normal development of the gut microbial community in dogs at an early age by avoiding a high fructose diet is hence crucial in preventing atopic diseases.^21^, ^24^, ^31^ Additionally, canned foods/fruits are the main source of bisphenol A (BPA), found in resins that coat the can's interior.^77^ One study found elevated concentrations of circulating BPA in dogs fed canned dog food for a short term which was associated with changes in the serum chemistry and alterations in the fecal microbiome.^78^

Mixed oils given to puppies as supplements was also significantly associated with increased AASS incidence later in life, in this study. Mixed oils mean that the dogs got a mixture of commercially available oils for humans and for pets and this food item is therefore composed of a mix of vegetable oils such as corn, soybean, and/or sunflower oils. They are poor in *n*‐3 PUFA, while rich in proinflammatory omega‐6 polyunsaturated fatty acids (*n*‐6 PUFA), such as linoleic acid (LA).^66^ The high intake of LA has been associated with a high prevalence of AD in Finnish and Australian children,^79^, ^80^ and high hay fever incidence in German children^81^ who consumed margarine. The impact of early life LA exposure on the development of future allergic diseases has been reported.^82^, ^83^ One study concluded that neonates that developed allergies also had higher LA concentrations in their umbilical cord lipids, and in their mothers' breast milk.^82^ The supposed biological mechanism linking *n*‐6 PUFA intake to allergic diseases involves eicosanoid mediators produced from the *n*‐6 PUFA arachidonic acid.^66^

Feeding dried animal parts during puppyhood was another significant potential risk factor for AASS incidence in adulthood. Dried animal parts are meat products processed under heat treatment for prolonged duration, until dry. The high temperature has a destructive effect on the nutrients, antioxidant, and digestive enzymes.^84^ The high temperature also causes protein denaturation and conjugation with other food components, which enhances its allergenicity.^85^ The Maillard reaction (MR, browning) is an interaction between the amino acids/cysteine and reduced sugars/ribose that occurs during heat processing. The MR produces multiple harmful substances such as heterocyclic amines.^86^ These MR compounds affect food digestibility, bioavailability, immunogenicity and subsequently allergenicity.^85^ They thus might enhance the incidence of IgE‐mediated allergies.^87^

Surprisingly, also drinking water from puddles during puppyhood was found to be significantly associated with increasing AASS later in life. Muddy water puddles are formed by rainwater on public roads and on footpaths.^88^ The enteropathogenic *Escherichia coli* (*E. coli*) O157 : H7 and pathogenic *Leptospira* persist in lake and puddle water more than in livestock drinking troughs or river water.^88^, ^89^ In studies evaluating gut microbiota dysbiosis levels, authors found more pathogenic *E. coli* in dogs with chronic diarrhea when compared to healthy dogs.^90^, ^91^ Gut microbial dysbiosis at an early age is a predisposing factor for future incidence of inflammatory and immune mediated diseases.^92^ Additionally, the contamination of puddle water with neonicotinoid insecticides and other pesticides in agricultural areas was reported as a risk for honey bees' intoxication.^93^ Therefore, we suggest that the exposure to pesticides in contaminated puddles could be a potential risk factor for intoxication for the livestock and dogs. However, in other studies we have seen that drinking from puddles has been negatively associated with disease, indicating that it might be beneficial from a hygiene hypothesis standpoint, so this also warrants further studies.

Eating carcasses outside during puppyhood was negatively associated with AASS development in adulthood (*P* = .6). Carcasses are defined as whole or pieces of fresh dead birds or animals^94^ to which dogs have access to. We assume that letting puppies eat carcasses outside mimics the dog's ancestral diet. It has been reported that the hypothetical canine ancestral diet consisted of fresh or recently killed animal carcasses or pieces of carcasses, carrion, meat scraps, bones, animal gut and others.^95^, ^96^, ^97^ An ancestral, species‐appropriate diet has been found to have a positive impact on chronic skin diseases.^24^ In addition, eating carcasses outside allows the puppies to swallow a dose of the soil microbiome adhered to the carcass, which could be viewed as a naturally occurring soil‐based probiotic. Indeed, a study on a murine model revealed that nonsterile soil consumption can contribute to a healthy gut microbial diversity to a parallel degree as the diet.^98^ This agrees with the hygiene hypothesis and with the microflora hypothesis.^8^, ^9^ This finding's lack of significance may be due to the low sample size for this variable and further investigation with a larger sample size is recommended.

The gut microbiome has a decisive role in modulating immune system maturation via crosstalk between the host's immunity and the microbiome.^99^ We thus assume that the more exposure to beneficial microbes during puppyhood, the more protection against AASS later in life.

### 4.3 Strengths and limitations

The current study has several strengths. The study data were obtained from a partially validated questionnaire,^13^ which provides reasonable and trusted data. The study took reverse causality into account by excluding the dogs under 1 year of age from analyses. Another strength of the study was the wide range of food items covered in the puppyhood food frequency questionnaire.

The current epidemiological study also has limitations. An owner reported food frequency questionnaire was used, which may have led to recall bias and misclassification of the food items. However, we have validated the owner's answers by resending the questionnaire for them to refill (A. Hielm‐Björkman, personal communication; A. HielmBjörkman, “Data validation” [email to M. Hemida], November 2, 2020, <anna.hielm-bjorkman@helsinki.fi>, [accessed November 2, 2020]). We can thus assume that recall bias was substantially reduced. Furthermore, the multiple analyses between a broad range of food groups and the outcome might result in findings that were significant only by chance. Moreover, the number of dogs included in the food variables categories were heterogeneous, and this might have caused some underestimation of potential risk or protective factors.

## 5 CONCLUSIONS

Our findings agree with the hygiene and the microflora hypothesis. We conclude that eating raw tripe, raw organ meats, fish oil supplements and human meal leftovers during puppyhood were identified as significant potential protective factors of AASS incidence. In contrast, eating fruits, mixed oil supplements, dried animal parts, and drinking from puddles outside during puppyhood were detected as significant potential risk factors of AASS incidence. These findings are further backed up by the diet ratio analysis where consumption of different feeding patterns during puppy age showed that even if the dog eats 80% of its food as dry, adding a minimum of 20% of the food as raw, significantly decreased the risk of AASS later in life. A concept of early exposure to beneficial bacteria by serving “real foods” and avoiding sugary fruits might be usable as an AASS prevention action. However, the study only suggests a causal relationship but does not prove it. Diet intervention studies are required to further elucidate the in‐depth association between dietary factors such as raw and dry foods, human meal leftovers and beneficial dosing of oils and the development of AASS.

## CONFLICT OF INTEREST DECLARATION

Authors declare no conflict of interest. The funders had no input on study design, data collection and analysis, decision to publish, or preparation of the manuscript.

## OFF‐LABEL ANTIMICROBIAL DECLARATION

Authors declare no off‐label use of antimicrobials.

## INSTITUTIONAL ANIMAL CARE AND USE COMMITTEE (IACUC) OR OTHER APPROVAL DECLARATION

DogRisk food frequency questionnaire was ethically approved at (29.4.2016) by Viikki campus ethical board, University of Helsinki.

## HUMAN ETHICS APPROVAL DECLARATION

Authors declare human ethics approval was not needed for this study.

## Supporting information

**Table S1**. Association between puppyhood food variables and incidence of owner‐reported allergy/atopy skin symptoms (AASS) in dogs above 1 year old.

**Table S2**. Supporting information.
