# Supplementary material for: Puppyhood diet as a factor in the development of owner‐reported allergy/atopy skin signs in adult dogs in Finland
Source: J Vet Intern Med. 2021 Jul 14;35(5):2374–83. doi: 10.1111/jvim.16211 (PMC8478020; doi:10.1111/jvim.16211)
Supplement: Supplementary file 1 — Table S1. Association between puppyhood food variables and incidence of owner‐reported allergy/atopy skin symptoms (AASS) in dogs above 1 year old. [file JVIM-35-2374-s002.pdf]

S1 Table. Association between puppyhood food variables and incidence of owner-reported allergy/atopy skin symptoms (AASS) in dogs above 1 year old.

Food items	Categories	4108 dogs population		P value	Multivariate logistic regression
		**Cases, % (n=762)**	**Controls, % (n=2346)**		
1. Berries	Never	51.8 (1165[a])	56.0 (506[b])	.10	Included
	A couple of times / year	23.1 (209[a])	25.3 (570[a])		
	A couple of times / month	15.3 (138[a])	16.5 (372[a])		
	A couple of times / week	4.9 (44[a])	4.7 (106[a])		
	Always or almost always	0.8 (7[a])	1.6 (36[a])		
2. Raw vegetables	Never	50.4 (465[a])	47.3 (1077[a])	.15	Included
	A couple of times / year	11.4 (105[a])	13.4 (304[a])		
	A couple of times / month	19.7 (182[a])	20.4 (465[a])		
	A couple of times / week	13.2 (122[a])	12.0 (274[a])		
	Always or almost always	5.3 (49[a])	6.9 (156[a])		
3. Cooked vegetables	Never	60.4 (559[a])	51.2 (1196[a])	**< .001**	Included
	A couple of times / year	9.0 (83[b])	11.3 (264[a])		
	A couple of times / month	16.2 (150[a])	15.8 (432[a])		
	A couple of times / week	9.8 (91[a])	11.9 (278[a])		
	Always or almost always	4.6 (43[b])	7.0 (164[a])		
4. Cooked potatoes	Never	48.4 (462[a])	46.7 (1097[a])	.06	Included
	A couple of times / year	23.2 (222[a])	22.9 (537[a])		
	A couple of times / month	18.0 (172[b])	21.1 (496[a])		
	A couple of times / week	9.2 (88[a])	7.4 (174[a])		
	Always or almost always	1.2 (11[a])	1.9 (45[a])		
5. Fruits	Never	53.2 (397[b])	57.6 (993[a])	**.01**	Included
	A couple of times / year	18.4 (137[a])	15.4 (266[a])		
	A couple of times / month	20.5 (153[b])	17.0 (293[a])		
	A couple of times / week	6.8 (51[a])	7.7 (132[a])		

	Always or almost always	1.1 (8[b])	2.3 (39[a])		
6. Raw tripe	Never	71.9 (668[a])	63.3 (1478[a])	**< .001**	Included
	A couple of times / year	5.9 (55[a])	7.0 (163[a])		
	A couple of times / month	12.9 (120[b])	15.8 (370[a])		
	A couple of times / week	7.5 (70[a])	9.4 (220[a])		
	Always or almost always	1.7 (16[b])	4.5 (105[a])		
7. Cooked tripe	Never	80.3 (736[a])	80.1 (1808[a])	.80	Not included
	A couple of times / year	7 (64[a])	8 (180[a])		
	A couple of times / month	7.3 (67[a])	7.2 (163[a])		
	A couple of times / week	3.7 (34[a])	3.3 (74[a])		
	Always or almost always	1.7 (16[a])	1.4 (32[a])		
8. Cooked organ meats	Never	75.8 (681b)	67.8 (1526[a])	**< .001**	Included
	A couple of times / year	7.6 (68b)	12.0 (269[a])		
	A couple of times / month	10.3 (93b)	13.3 (300[a])		
	A couple of times / week	4.4 (40a)	5.3 (119[a])		
	Always or almost always	1.9 (17a)	1.6 (36[a])		
9. Raw organ meats	Never	84.7 (752[b])	77.3 (1697[a])	**< .001**	Included
	A couple of times / year	3.2 (28[b])	5.7 (126[a])		
	A couple of times / month	7.5 (67[b])	10.1 (221[a])		
	A couple of times / week	3.5 (31[a])	5.1 (111[a])		
	Always or almost always	1.1 (10[a])	1.8 (40[a])		
10. Raw bone and cartilage	Never	44.0 (433[b])	36.0 (881[a])	**< .001**	Included
	A couple of times / year	12.3 (121[b])	15.7 (385[a])		
	A couple of times / month	24.3 (239[a])	25.5 (623[a])		
	A couple of times / week	13.2 (130[a])	15.1 (369[a])		
	Always or almost always	6.2 (61[a])	7.7 (188[a])		
11. Cooked bone and cartilage	Never	51.6 (502[b])	47.7 (1148[a])	.13	Included
	A couple of times / year	15.3 (149[a])	18.0 (434[a])		
	A couple of times / month	20.7 (201[a])	22.8 (549[a])		
	A couple of times / week	10.2 (99[a])	9.3 (224[a])		

	Always or almost always	2.2 (21[a])	2.1 (50[a])		
12. Sour milk products	Never	0.5 (4[a])	0.2 (5[a])	**.02**	Included
	A couple of times / year	10.7 (91[b])	8.0 (169[a])		
	A couple of times / month	31.1 (264[a])	28.7 (607[a])		
	A couple of times / week	35.2 (299[a])	36.4 (771[a])		
	Always or almost always	22.6 (192[b])	26.7 (565[a])		
13. Other milk products	Never	57.2 (549[a])	52.5 (1260[a])	.09	Included
	A couple of times / year	28.7 (275[a])	30.8 (738[a])		
	A couple of times / month	10.9 (105[a])	12.2 (293[a])		
	A couple of times / week	2.3 (22[a])	3.2 (76[a])		
	Always or almost always	0.8 (8[a])	1.3 (31[a])		
14. Cheese	Never	0.4 (2[a])	0.6 (8[a])	.65	Not included
	A couple of times / year	28.7 (158[a])	29.9 (395[a])		
	A couple of times / month	46.2 (254[a])	46.7 (618[a])		
	A couple of times / week	21.6 (119[a])	19.0 (252[a])		
	Always or almost always	3.1 (17[a])	3.8 (50[a])		
15. Vegetable oils	Never	33.3 (323[a])	27.8 (657[a])	**.001**	Included
	A couple of times / year	7.9 (77[b])	11.8 (280[a])		
	A couple of times / month	20.4 (198[a])	22.4 (529[a])		
	A couple of times / week	22.7 (220[a])	22.0 (521[a])		
	Always or almost always	15.8 (153[a])	16.0 (379[a])		
16. Fish oil	Never	65.8 (586[a])	62.9 (1369[a])	**< .001**	Included
	A couple of times / year	5.8 (52[b])	8.4 (182[a])		
	A couple of times / month	11.3 (101[b])	15.1 (329[a])		
	A couple of times / week	10.4 (93[a])	9.1 (199[a])		
	Always or almost always	6.6 (59[b])	4.4 (96[a])		
17. Mixed oils	Never	86.5 (643[b])	91.6 (1605[a])	**< .001**	Included
	A couple of times / year	2.6 (19[a])	2.2 (38[a])		
	A couple of times / month	2.8 (21[a])	2.7 (47[a])		
	A couple of times / week	2.6 (19[a])	1.9 (34[a])		

	Always or almost always	5.5 (41[b])	1.7 (29[a])		
18. Animal fat	Never	82.0 (579[b])	73.7 (1222[a])	**< .001**	Included
	A couple of times / year	6.7 (47[a])	8.4 (140[a])		
	A couple of times / month	5.8 (41[b])	9.6 (159[a])		
	A couple of times / week	4.5 (32[a])	5.6 (92[a])		
	Always or almost always	1.0 (7[b])	2.7 (44[a])		
19. Raw eggs	Never	55.2 (523[b])	48.2 (1138[a])	**< .001**	Included
	A couple of times / year	11.7 (111[b])	15.5 (367[a])		
	A couple of times / month	22.3 (211[a])	22.4 (528[a])		
	A couple of times / week	9.9 (94[a])	11.5 (271[a])		
	Always or almost always	0.9 (9[b])	2.4 (57[a])		
20. Cooked eggs	Never	57.7 (532[b])	48.4 (1114[a])	**< .001**	Included
	A couple of times / year	16.4 (151[a])	18.3 (421[a])		
	A couple of times / month	18.0 (166[b])	24.0 (552[a])		
	A couple of times / week	5.7 (53[a])	7.2 (166[a])		
	Always or almost always	2.2 (20[a])	2.2 (50[a])		
21. Dry food	Never	3.4 (39[a])	4.6 (130[a])	**.02**	Included
	A couple of times / year	1.0 (12[a])	1.5 (41[a])		
	A couple of times / month	1.0 (12[a])	1.8 (50[a])		
	A couple of times / week	4.8 (55[a])	6.3 (178[a])		
	Always or almost always	89.7 (1026[b])	85.8 (2416[a])		
22. Wet food[*]	Never	55.7 (549[a])	53.4 (1280[a])	.48	Not included
	A couple of times / year	13.0 (128[a])	15.0 (359[a])		
	A couple of times / month	17.5 (172[a])	18.0 (432[a])		
	A couple of times / week	9.5 (94[a])	8.8 (211[a])		
	Always or almost always	4.3 (42[a])	4.7 (113[a])		
23. Medical dry food	Never	94.5 (812[b])	99.0 (2088[a])	**< .001**	Included
	A couple of times / year	0.8 (7[a])	0.3 (7[a])		
	A couple of times / month	0.8 (7[b])	0.2 (4[a])		
	A couple of times / week	1.0 (9[b])	0.04 (1[a])		

	Always or almost always	2.8 (24[b])	0.5 (10[a])		
24. Blood crepes	Never	83.9 (762[b])	74.9 (1681[a])	**< .001**	Included
	A couple of times / year	8.6 (78[b])	14.5 (326[a])		
	A couple of times / month	5.7 (52[b])	8.6 (194[a])		
	A couple of times / week	1.7 (15[a])	1.7 (38[a])		
	Always or almost always	0.1 (1[a])	0.2 (5[a])		
25. Liver casserole	Never	42.6 (423[b])	34.9 (860[a])	**< .001**	Included
	A couple of times / year	23.3 (231[b])	26.9 (662[a])		
	A couple of times / month	27.0 (268[a])	28.7 (707[a])		
	A couple of times / week	6.3 (63[b])	8.6 (213[a])		
	Always or almost always	0.8 (8[a])	0.9 (22[a])		
26. Dried fish	Never	80.5 (711[a])	79.2 (1740[a])	.09	Included
	A couple of times / year	9.2 (81[b])	12.0 (263[a])		
	A couple of times / month	7.4 (65[a])	7.0 (153[a])		
	A couple of times / week	2.7 (24[a])	1.7 (38[a])		
	Always or almost always	0.2 (2[a])	0.1 (3[a])		
27. Human meal leftovers	Never	31.9 (314[b])	21.2 (527[a])	**< .001**	Included
	A couple of times / year	23.1 (227[a])	22.5 (561[a])		
	A couple of times / month	26.1 (257[a])	29.2 (728[a])		
	A couple of times / week	14.7 (145[b])	19.6 (489[a])		
	Always or almost always	4.2 (41[b])	7.5 (186[a])		
28. Cooked porridge	Never	26.4 (262[b])	20.0 (500[a])	**< .001**	Included
	A couple of times / year	22.1 (219[b])	18.7 (466[a])		
	A couple of times / month	24.9 (247[b])	29.5 (738[a])		
	A couple of times / week	17.5 (174[a])	19.7 (492[a])		
	Always or almost always	9.1 (90[b])	12.1 (302[a])		
29. Cooked rice	Never	14.9 (152[a])	13.9 (349[a])	.83	Not included
	A couple of times / year	18.3 (186[a])	18.1 (455[a])		
	A couple of times / month	35.0 (356[a])	34.4 (864[a])		
	A couple of times / week	20.6 (210[a])	21.3 (534[a])		

	Always or almost always	11.1 (113[a])	12.2 (306[a])		
30. Treats	Never	9.6 (98[a])	9.2 (236[a])	.05	Included
	A couple of times / year	12.2 (125[a])	13.8 (354[a])		
	A couple of times / month	26.5 (272[a])	29.7 (761[a])		
	A couple of times / week	38.0 (390[b])	33.1 (848[a])		
	Always or almost always	13.7 (141[a])	14.2 (365[a])		
31. Rawhides	Never	4.7 (49[b])	6.5 (170[a])	**.04**	Included
	A couple of times / year	9.6 (100[a])	11.4 (297[a])		
	A couple of times / month	28.1 (294[a])	28.3 (736[a])		
	A couple of times / week	36.1 (378[a])	35.0 (911[a])		
	Always or almost always	21.6 (226[a])	18.8 (488[a])		
32. Raw poultry	Never	79.3 (411[a])	79.0 (874[a])	.99	Not included
	A couple of times / year	1.4 (7[a])	1.6 (18[a])		
	A couple of times / month	5.0 (26[a])	5.2 (58[a])		
	A couple of times / week	8.1 (42[a])	7.8 (86[a])		
	Always or almost always	6.2 (32[a])	6.3 (70[a])		
33. Cooked poultry	Never	36.3 (348[a])	35.5 (847[a])	.55	Not included
	A couple of times / year	9.7 (93[a])	11.7 (278[a])		
	A couple of times / month	27.5 (264[a])	26.4 (628[a])		
	A couple of times / week	19.5 (187[a])	19.8 (472[a])		
	Always or almost always	7.0 (67[a])	6.6 (158[a])		
34. Raw red meat	Never	40.0 (362[a])	33.1 (718[a])	**.002**	Included
	A couple of times / year	6.0 (54[a])	7.8 (169[a])		
	A couple of times / month	21.9 (198[a])	21.6 (469[a])		
	A couple of times / week	18.3 (166[b])	21.9 (475[a])		
	Always or almost always	13.8 (125[a])	15.7 (340[a])		
35. Cooked red meat	Never	44.2 (363[a])	44.8 (884[a])	.51	Not included
	A couple of times / year	6.7 (55[a])	8.3 (164[a])		
	A couple of times / month	23.5 (193[a])	21.3 (421[a])		
	A couple of times / week	18.3 (150[a])	18.3 (361[a])		

	Always or almost always	7.3 (60[a])	7.2 (142[a])		
36. Processed meat	Never	19.0 (194[a])	17.0 (429[a])	.49	Not included
	A couple of times / year	20.2 (206[a])	21.0 (528[a])		
	A couple of times / month	35.5 (362[a])	35.0 (882[a])		
	A couple of times / week	22.1 (225[a])	24.1 (606[a])		
	Always or almost always	3.2 (33[a])	2.9 (72[a])		
37. Raw fish	Never	79.6 (716[b])	75.3 (1675[a])	.09	Included
	A couple of times / year	4.8 (43[b])	6.7 (148[a])		
	A couple of times / month	9.8 (88[a])	11.3 (251[a])		
	A couple of times / week	5.0 (45[a])	5.5 (123[a])		
	Always or almost always	0.8 (7[a])	1.2 (26[a])		
38. Cooked fish	Never	54.3 (503[b])	46.0 (1062[a])	**< .001**	Included
	A couple of times / year	15.7 (145[b])	18.6 (430[a])		
	A couple of times / month	23.3 (216[b])	27.5 (636[a])		
	A couple of times / week	5.8 (54[a])	7.3 (169[a])		
	Always or almost always	0.9 (8[a])	0.6 (13[a])		
39. Eating sticks outside	Never	16.1 (162[b])	21.4 (530[a])	**< .001**	Included
	A couple of times / year	16.9 (170[a])	18.6 (460[a])		
	A couple of times / month	26.1 (262[a])	25.6 (635[a])		
	A couple of times / week	30.7 (309[b])	22.9 (566[a])		
	Always or almost always	10.1 (102[a])	11.5 (286[a])		
40. Eating grass outside	Never	10.0 (101[a])	9.0 (229[a])	.83	Not included
	A couple of times / year	18.4 (187[a])	19.8 (501[a])		
	A couple of times / month	34.4 (349[a])	34.5 (876[a])		
	A couple of times / week	27.2 (276[a])	26.5 (671[a])		
	Always or almost always	10.0 (102[a])	10.2 (259[a])		
41. Eating clay and stones outside	Never	64.3 (597[a])	66.8 (1529[a])	.31	Not included
	A couple of times / year	15.8 (147[a])	14.9 (340[a])		
	A couple of times / month	11.4 (106[a])	9.3 (214[a])		
	A couple of times / week	6.3 (58[a])	6.2 (143[a])		

	Always or almost always	2.2 (20[a])	2.8 (63[a])		
42. Eating dirt outside	Never	54.0 (506[a])	57.7 (1347[a])	**.01**	Included
	A couple of times / year	25.1 (235[b])	21.7 (507[a])		
	A couple of times / month	13.2 (124[a])	12.0 (280[a])		
	A couple of times / week	6.8 (64[a])	6.5 (151[a])		
	Always or almost always	0.9 (8[b])	2.1 (50[a])		
43. Drinking from puddles outside	Never	11.6 (118[a])	10.6 (268[a])	**.001**	Included
	A couple of times / year	20.3 (206[a])	18.0 (457[a])		
	A couple of times / month	32.1 (325[a])	29.3 (743[a])		
	A couple of times / week	26.8 (272[a])	28.3 (716[a])		
	Always or almost always	9.2 (93[b])	13.8 (349[a])		
44. Eating feces outside	Never	39.0 (379[a])	40.6 (972[a])	**.02**	Included
	A couple of times / year	22.3 (217[a])	22.2 (531[a])		
	A couple of times / month	21.3 (207[a])	20.2 (483[a])		
	A couple of times / week	11.5 (112[a])	13.5 (323[a])		
	Always or almost always	5.9 (57[b])	3.6 (85[a])		
45. Eating carcasses outside	Never	70.0 (651[b])	61.9 (1444[a])	**< .001**	Included
	A couple of times / year	20.2 (188[b])	23.4 (547[a])		
	A couple of times / month	7.5 (70[b])	11.1 (259[a])		
	A couple of times / week	1.9 (18[a])	2.8 (66[a])		
	Always or almost always	0.3 (3[a])	0.7 (17[a])		
46. Dried animal parts	Never	13.2 (136[a])	14.1 (356[a])	.10	Included
	A couple of times / year	12.1 (124[b])	15.1 (382[a])		
	A couple of times / month	41.8 (430[a])	40.6 (1027[a])		
	A couple of times / week	29.1 (299[a])	26.3 (665[a])		
	Always or almost always	3.9 (40[a])	3.8 (97[a])		

[a,b] Means the different superscripts are significantly different within the same row ($P < .05$). [*]: refers to canned or tinned food, plastic wrapped dog wet food, and other forms of dog wet food in bags. Bolded: significant p-value < .05.
