# Supplementary material for: Puppyhood diet as a factor in the development of owner‐reported allergy/atopy skin signs in adult dogs in Finland
Source: J Vet Intern Med. 2021 Jul 14;35(5):2374–83. doi: 10.1111/jvim.16211 (PMC8478020; doi:10.1111/jvim.16211)
Supplement: Supplementary file 2 — Table S2. Supporting information. [file JVIM-35-2374-s001.pdf]

The following question from the DogRisk FFQ is used in the current study (only in Finnish language: www.ruokintakysely.fi)

Question 21.* Has your dog had any of the following diseases? Select 1-4 ticks per line + starting age, if it has/had the disease

**Diseases**	Has your dog had it?¥		How often ?		It started at the age of		Does it still have the disease?	The disease disappeared after changing the diet	I have not noticed that a diet change would have helped
	Yes	No	Rarely	Often	Years	Months			
Ear inflammation	☐	☐	☐	☐	Choose an item.	Choose an item.	☐	☐	☐
Dermatitis (eg rash, hot-spot)	☐	☐	☐	☐	Choose an item.	Choose an item.	☐	☐	☐
Demodicosis	☐	☐	☐	☐	Choose an item.	Choose an item.	☐	☐	☐
Inflammation between the toes (= furunculosis)	☐	☐	☐	☐	Choose an item.	Choose an item.	☐	☐	☐
"Allergy", atopy, (skin-symptoms)©	☐	☐	☐	☐	Choose an item.	Choose an item.	☐	☐	☐
Acanthosis nigricans	☐	☐	☐	☐	Choose an item.	Choose an item.	☐	☐	☐
Seborrhea	☐	☐	☐	☐	Choose an item.	Choose an item.	☐	☐	☐

*: It is a table-form question asking for the health status of the dog, including 117 diseases. ©: The question used in the current study. ¥: The dichotomous variable used as the main dependent variable in the study.
